# Comparison of SARS-CoV-2 IgG responses in hemodialysis patients and healthcare workers after COVID-19 vaccination

**DOI:** 10.3389/fimmu.2025.1586468

**Published:** 2025-07-15

**Authors:** Hakkı Öztürk, Metin Özsoy, Ayşegül Tuna, Artuner Varlibas, Salih Cesur, Altan Aksoy, Aydın Çifci, Mehmet Emin Demir

**Affiliations:** ^1^ Infectious Diseases Epidemiologist, Dialysis Physician, Private Ankara Dialysis Center, Ankara, Türkiye; ^2^ Clinic of Infectious Diseases and Clinical Microbiology, University of Health Sciences, Ankara Training and Research Hospital, Ankara, Türkiye; ^3^ Department of Internal Medicine, Faculty of Medicine, Kırıkkale University, Kırıkkale, Türkiye; ^4^ Department of Medical Microbiology, Ankara Training and Research Hospital, University of Health Sciences, Ankara, Türkiye; ^5^ Atılım University, School of Medicine, Department of Nephrology, Ankara, Türkiye

**Keywords:** SARS-CoV-2, IgG antibody, hemodialysis, COVID-19 vaccination, immune response

## Abstract

**Aim:**

This study aimed to compare SARS-CoV-2 IgG antibody levels in hemodialysis (HD) patients and healthcare workers (HCWs) after COVID-19 vaccination and to identify factors influencing these levels.

**Materials and methods:**

A total of 193 participants were included: 104 HD patients and 89 age- and sex-matched HCWs as controls. All had completed a primary COVID-19 vaccination series (two doses of CoronaVac or BNT162b2) and a booster dose. SARS-CoV-2 anti-spike IgG was measured at least one month after the last vaccine dose using a commercial immunoassay (Abbott SARS-CoV-2 IgG II Quant, CMIA). Results in Arbitrary Units (AU/mL) were converted to WHO standard Binding Antibody Units (BAU/mL) (1 AU/mL = 0.142 BAU/mL). IgG titers ≥7.1 BAU/mL (equivalent to 50 AU/mL) were considered positive.

**Results:**

All participants had positive SARS-CoV-2 IgG antibodies. There were no statistically significant differences in IgG levels between HD patients and HCWs at any individual time interval (<3 months, 3–6 months, or >6 months) or in the overall mean titers (HD: 1259 ± 1112 BAU/mL; HCW: 1002 ± 765 BAU/mL; p = 0.216). No individual in either group had an IgG titer below 7.1 BAU/mL. Vaccine type, dialysis vintage, and presence of comorbidities did not significantly impact antibody levels. In the HCWs group, those vaccinated only with CoronaVac had significantly lower IgG levels than those receiving only BNT162b2 or a heterologous regimen (CoronaVac followed by BNT162b2). However, among HD patients, IgG levels did not differ by vaccine regimen.

**Conclusion:**

HD patients mounted a SARS-CoV-2 IgG antibody response comparable to that of healthy HCWs, with no participant falling below the positivity threshold. Dialysis duration and comorbid conditions did not significantly affect post-vaccination IgG levels. While HCWs who received only CoronaVac showed lower antibody titers than those who received BNT162b2 or a heterologous schedule, this difference was not observed in HD patients. These results suggest that COVID-19 vaccination elicits a robust humoral immune response in the HD population, underscoring the benefit of vaccination in this high-risk group.

## Introduction

The COVID-19 pandemic caused by SARS-CoV-2 has led to significant mortality and morbidity worldwide and in Turkey (1–3). As of May 2025, there have been approximately 704 million confirmed COVID-19 cases globally, resulting in about 7 million deaths (2). In Turkey, about 17.2 million cases have been reported, with a total of ~102,000 deaths (3). Globally, ~16.4 billion people have received at least one dose of a COVID-19 vaccine (4).

Hemodialysis (HD) patients are a high-risk group for COVID-19 infection and have high mortality rates (5–8). In HD patients, male sex and underlying cardiovascular or respiratory diseases, diabetes, and cardiovascular disease are significant risk factors for COVID-19 mortality (9–11).

COVID-19 vaccines have been shown to reduce mortality and morbidity in HD patients (9, 10). During the pandemic, vaccines were developed within one year and deployed globally under emergency use authorization (12). In Turkey, two vaccines were predominantly used: the inactivated viral vaccine CoronaVac (Sinovac, China) and the mRNA vaccine BNT162b2 (Pfizer-BioNTech, USA/Germany). Both were approved for use and widely administered in mass vaccination programs. HD patients were prioritized for vaccination, and a booster dose was recommended given concerns about their potentially attenuated immune response (13). Although HD patients may have weaker immune responses compared to healthy individuals and may respond suboptimally to vaccines, studies have demonstrated that even immunocompromised patients can develop preventive antibody responses similar to those of healthy individuals, as reported by Venerito et al. in a psoriatic arthritis cohort receiving active immunosuppressive therapy (14, 15).

Several studies have indicated that SARS-CoV-2 antibody levels may decline more rapidly in HD patients due to weaker humoral and cellular immune responses (16, 17). However, booster vaccinations have been shown to significantly enhance antibody titers in this population (18, 19). Data regarding the durability of vaccine-induced antibody responses in HD patients and influencing factors (such as vaccine type, prior COVID-19 infection, and time elapsed since vaccination) remain limited. Thus, it is crucial to determine whether HD patients achieve adequate antibody protection comparable to healthy individuals, and how long this protection persists, to optimize booster timing and mitigate infection risk.

The COVID-19 pandemic has significantly impacted HCWs, causing substantial morbidity and mortality. Various vaccination strategies have been implemented by health systems during different pandemic waves. A large European cohort study involving 63,516 healthcare workers reported a breakthrough COVID-19 infection rate of approximately 20% following booster doses, emphasizing the importance of timely boosters based on declining antibody levels (20). Comparative analyses from Italy (retrospective observational study involving 6,030 healthcare workers across three pandemic waves) and Brazil (cross-sectional descriptive study including 368 individuals from the general population across two waves) also demonstrated important variations in infection rates, influenced significantly by the circulating variants. In Italy, the highest positivity rate was observed during the second wave (5.4%), while in Brazil, positivity rates notably increased during the Omicron wave (56% in late 2022) despite high vaccination coverage (21, 22). These findings collectively underline the importance of booster strategies and sustained monitoring of protective antibody levels, particularly among high risk patients such as HCWs and immunocompromised individuals, such as HD patients.

In this study, we compared the SARS-CoV-2 anti-spike IgG antibody levels of HD patients to those of healthcare workers (HCWs) after completion of primary COVID-19 vaccination and a booster dose. We also analyzed the influence of vaccine type (CoronaVac vs BNT162b2, or a heterologous combination) and prior SARS-CoV-2 infection on antibody levels. An important aspect of our analysis was accounting for the interval between the last vaccine dose and antibody measurement, to evaluate waning immunity over time. We hypothesized that, despite immunosuppressive factors, HD patients who are appropriately vaccinated would have antibody levels comparable to healthy controls. We also aimed to identify any subgroups with reduced responses (e.g., those with only inactivated vaccine or with certain comorbidities) to inform vaccination strategies for this vulnerable population.

## Methods

### Study design and participants

This single-center observational study included 104 adult HD patients receiving maintenance dialysis at a private dialysis center in Ankara, Turkey, and 89 adult HCWs from the same region who served as the control group. The control group was frequency-matched to the HD group by age and sex. All participants had completed a primary two-dose COVID-19 vaccination series with either CoronaVac or BNT162b2, followed by a booster dose with either the same or the other vaccine (i.e., some received three homologous doses and others a heterologous regimen). Participants were recruited at least one month after their most recent COVID-19 vaccine dose. Individuals with active COVID-19 infection or on immunosuppressive therapies were excluded. Prior COVID-19 infection status was determined by clinical history and documented positive PCR tests (if available).

### Ethical approval

The study was approved by the Bilkent City Hospital Ethics Committee (Approval No: E2-22-XX) and conducted in accordance with the Declaration of Helsinki. All participants provided written informed consent.

### Data collection

Demographic data (age, sex), clinical data (comorbidities such as hypertension, diabetes mellitus, and dyslipidemia; dialysis duration; chronic kidney disease stage), and vaccination details (vaccine type for each dose, total number of doses) were obtained from medical records and participant interviews. The time interval between the last vaccine dose and the blood sample collection (in days) was recorded for each participant. History of COVID-19 infection was noted (yes/no).

### Laboratory methods

Blood samples were collected from all participants to measure SARS-CoV-2 spike protein-specific IgG antibody levels. The SARS-CoV-2 IgG titers were quantified using the Abbott AdviseDx SARS-CoV-2 IgG II Quant assay (Abbott Laboratories, USA) on the ARCHITECT i system. This is a chemiluminescent microparticle immunoassay (CMIA) that detects IgG antibodies against the receptor-binding domain (RBD) of the spike protein. Testing was performed in the hospital’s microbiology laboratory according to the manufacturer’s instructions. The assay provides results in arbitrary units per milliliter (AU/mL), which were converted to WHO International Standard binding antibody units per milliliter (BAU/mL) using the conversion factor 1 AU/mL = 0.142 BAU/mL (23). This conversion aligns the results with the First WHO International Standard for anti-SARS-CoV-2 immunoglobulin. For analysis, we considered the positivity cutoff of the assay as 50 AU/mL, which corresponds to 7.1 BAU/mL. IgG levels ≥7.1 BAU/mL were interpreted as positive. All samples in this study were above this threshold.

### Outcome measures

The primary outcome was the SARS-CoV-2 anti-spike IgG concentration (in BAU/mL) measured in HD patients versus controls. Secondary outcomes included comparisons of IgG levels by vaccine type (only CoronaVac vs only BNT162b2 vs heterologous) and by prior COVID-19 infection status, as well as the relationship between IgG levels and time since last vaccination.

### Statistical analysis

Continuous variables were expressed as means ± standard deviation (SD). Categorical variables were summarized using counts and percentages. Normality was assessed using the Shapiro–Wilk test. Non-normally distributed data were compared using the Mann–Whitney U test for two groups and Kruskal–Wallis test with Dunn’s *post-hoc* correction for multiple groups. Two-way ANOVA was performed on log-transformed antibody titers to test for main effects and interactions between participant groups (HD vs HCW) and prior infection status or vaccine regimen. Exponential decay models were fitted to antibody titers using non-linear least squares regression analysis. Differences in antibody waning rates were analyzed using linear regression with group-by-time interaction terms. Multivariate linear regression analyses were used to control for confounding variables. A two-tailed alpha of 0.05 was considered statistically significant. Statistical analyses were performed using SPSS version 26.0 (IBM Corp., Armonk, NY, USA).

## Results

### Participant characteristics

The demographic and clinical characteristics of the HD patients and control HCWs are summarized in **Table 1**.

**Table 1:** Baseline demographic and clinical characteristics of hemodialysis patients and healthcare workers.

Characteristic	HD patients (N=104)	HCWs (N=89)	p-value
Age, years	53 ± 27	39 ± 8	<0.001
Male sex, n (%)	61 (58.7%)	40 (44.9%)	0.057
Hypertension, n (%)	45 (43.2%)	5 (5.6%)	<0.001
Diabetes mellitus, n (%)	37 (35.5%)	3 (3.3%)	<0.001
CVD, n (%)	24 (23.0%)	2 (2.2%)	<0.001
Time since last dose, days	88 ± 62	81 ± 69	0.866
< 3 months, n (%)	45 (43.1%)	40 (44.9%)	–
3–6 months, n (%)	52 (50.0%)	43 (48.3%)	–
> 6 months, n (%)	7 (6.9%)	6 (6.8%)	–

CKD, chronic kidney disease; HD, hemodialysis; HCWs, healthcare workers.

The HD group had a mean age of 59.4 ± 10.6 years (median 64), which was significantly higher than the control group’s mean age of 39.9 ± 11.6 years (median 42; p<0.001). The HD patients were 61 (58.7%) male, while the HCW group was 40 (44.9%) male; this difference in sex distribution had a border statistically significant (p= 0.057). As expected, HD patients had a high burden of comorbid conditions. Hypertension was present in 43.2% of HD patients, compared to 5.6% of controls (p<0.001). Similarly, diabetes mellitus was more frequent in HD patients (35.5% vs 3.3% in controls, p<0.001), as was cardiovascular disease (23.0% vs 2.2%, p= 0.001). A small proportion of participants had a history of prior COVID-19 infection: 5 (4.8%) in the HD group vs 22 (24.7%) in the control group (p < 0.001). The interval from the last vaccine dose to antibody measurement was similar between groups. The mean time since last dose was 88 ± 62 days for HD patients and 86 days 81 ± 69 for HCWs (p= 0.866). Thus, aside from age and comorbidities (which were more prevalent in HD patients), the groups were comparable in terms of sex and vaccination timing.

Vaccination details are presented in **Table 2**. All participants received a total of three COVID-19 vaccine doses except for a few controls who had only two doses. In the HD group, 29 patients (27.9%) received only CoronaVac for all doses, 34 (32.7%) received only BNT162b2, and 41 (39.4%) received a heterologous regimen (primarily two doses of CoronaVac followed by a BNT162b2 booster). In the control group, 12 individuals (13.5%) received only CoronaVac, 27 (30.3%) received only BNT162b2, and 50 (56.2%) received the heterologous regimen. The distribution of vaccine regimens differed significantly between the two groups (χ² test p= 0.022). Notably, a heterologous schedule was more common among HCWs (56.2%) than HD patients (39.4%), while HD patients were more likely to have received only CoronaVac or only BNT162b2 (**Table 2**). This likely reflects that HCWs had greater access to or preference for the mRNA booster. The total number of vaccine doses was similar between groups (mean ~3.4 doses in HD vs 3.3 in HCWs).

**Table 2:** Distribution of COVID-19 vaccination regimens among hemodialysis patients and healthcare workers.

Vaccination regimen	HD patients (N=104)	HCWs (N=89)	p-value
2 doses CoronaVac	0	2 (2.2%)	0.022
3 doses CoronaVac	29 (30%)	10 (11.2%)	
2 doses BNT162b2	0 (10%)	3 (3.3%)	
3 doses BNT162b2	34 (0%)	24 (26.9%)	
Heterologous regimen*	41 (40%)	50 (56.2%)	

* Primarily two doses of CoronaVac followed by a BNT162b2 booster. HD; hemodialysis patients, HCWs; healthcare workers

**Table 3:** IgG levels (BAU/mL) according to time since last dose.

Groups	Vaccine types, n	< 3months	3-6 months	> 6 months	Mean*
HD patients	CoronaVac, n=29 BNT162b, n=34 Heterologous regimen, n= 41	1919 ± 1209 1488 ± 758 2018 ± 1233	1388 ± 823 1052 ± 725 1360 ± 812	782 ± 578 865 ± 502 1190 ± 782	1340.22 ± 824.67
HCWs	CoronaVac, n=12 BNT162b, n= 27 Heterologous regimen, n=50	876 ± 547 1825 ± 541 916 ± 705	684 ± 490 1290 ± 780 875 ± 690	648 ± 466 825 ± 603 798 ± 493	970.78 ± 590.56

*The mean SARS-CoV-2 IgG levels (calculated as the overall mean of all measurements for each group) showed no statistically significant difference between HD patients and HCWs (p = 0.720). HD; hemodialysis patients, HCW; healthcare workers.

### Humoral immune response (IgG levels)

**Figure 1:**
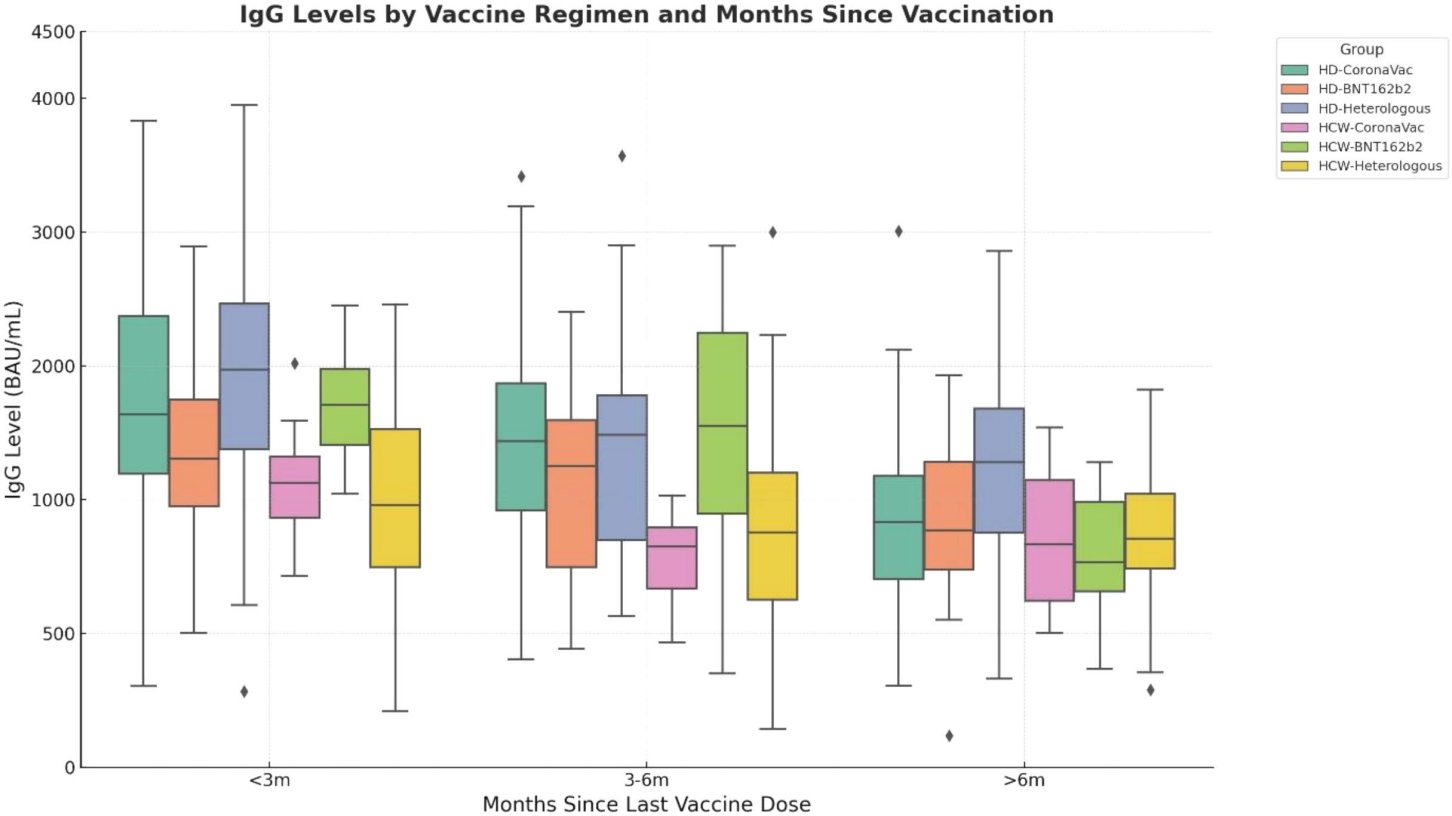
Trends in SARS-CoV-2 anti-spike IgG antibody levels (BAU/mL) over time according to vaccine type. Initial antibody levels were highest in recipients of BNT162b2, lowest in those vaccinated with CoronaVac, and intermediate among participants with the heterologous regimen; however, these differences were not statistically significant (p>0.05, differences between groups were assessed using the Kruskal–Wallis test). All vaccine regimens showed similar rates of antibody decline, highlighting a consistent waning pattern irrespective of vaccine type.

Despite a gradual decrease in antibody titers over time, there were no statistically significant differences in SARS-CoV-2 anti-spike IgG levels between HD patients and HCWs at any individual time interval (<3 months, 3–6 months, or >6 months); p>0.05 or when comparing overall mean titers (HD: 1340.22 ± 824.67 BAU/mL; HCW: 970.78 ± 590.56 BAU/mL; p = 0.720; **Table 3**). **Figure 1** illustrates trends in antibody titers by vaccine type. Although initial antibody titers were highest with BNT162b2, lowest with CoronaVac, and intermediate with the heterologous regimen, these differences were not statistically significant (p=0.089). Importantly, the rate of antibody decline was consistent across vaccine types and participant groups.

**Figure 2:**
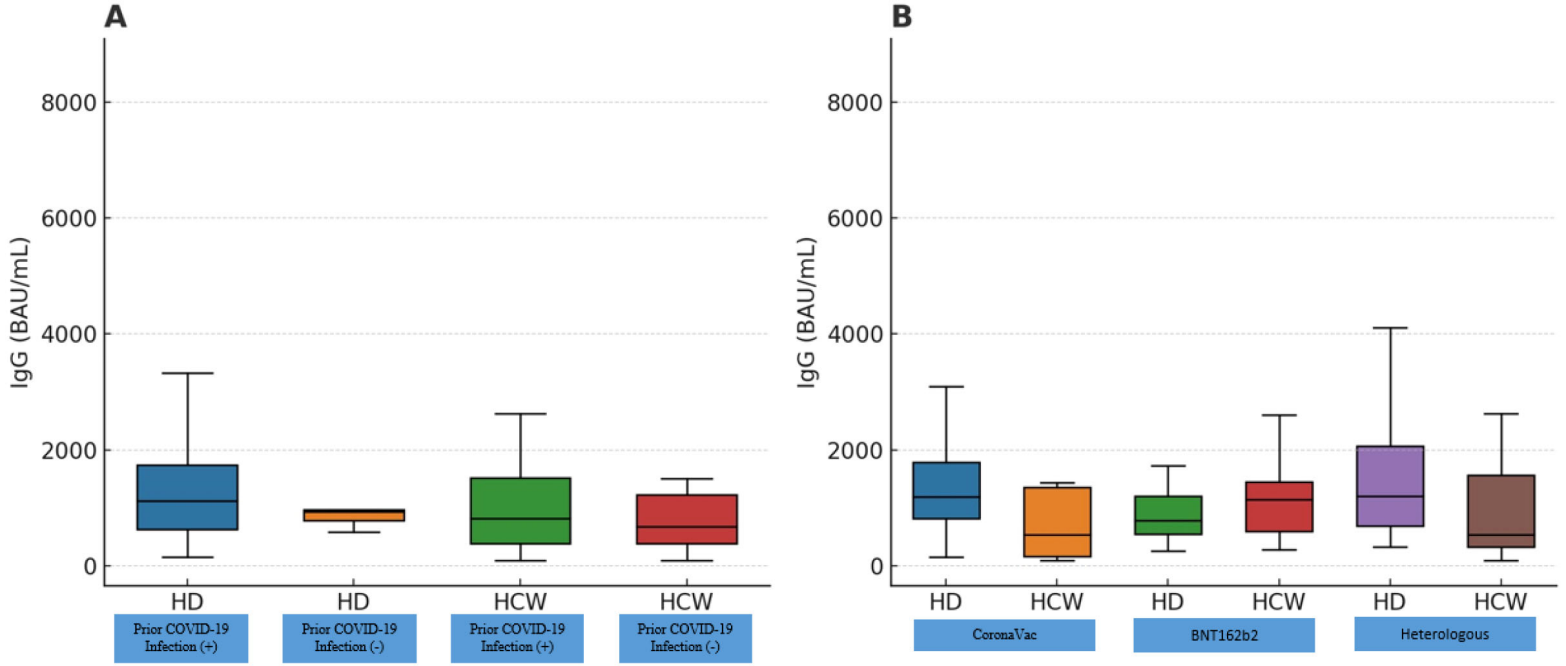
Mean SARS-CoV-2 anti-spike IgG antibody levels (BAU/mL), calculated from all measurements presented in **Table 2** for each group. In **(A)** antibody titers are compared between hemodialysis (HD) patients and healthcare workers (HCWs), stratified by the presence or absence of prior PCR-confirmed COVID-19 infection. In **(B)** groups are categorized according to vaccine regimen: CoronaVac-only, BNT162b2-only, or a heterologous regimen (CoronaVac followed by BNT162b2). Each data point represents a group’s mean IgG level; boxplots illustrate medians and interquartile ranges (IQR). HD patients achieved antibody levels comparable to HCWs, and prior infection did not significantly elevate antibody titers in either group (p > 0.05, for all comparisons). Among HCWs, those vaccinated exclusively with CoronaVac exhibited significantly lower IgG levels compared to those receiving only BNT162b2 (p = 0.035) or the heterologous regimen (p = 0.004). In contrast, no significant differences between vaccine regimens were observed among HD patients (p>0.05). HD, Hemodialysis patients; HCW, Healthcare workers.

**Figure 2** compares mean IgG levels calculated from **Table 3**, stratified by prior infection (Panel A) and vaccine regimen (Panel B). Prior PCR-confirmed COVID-19 infection did not significantly influence antibody titers in either HD or HCW groups (p = 0.256 vs p = 0.571). Among HCWs, IgG levels were significantly lower in those vaccinated exclusively with CoronaVac compared to BNT162b2 alone (p=0.008) and marginally lower compared to the heterologous regimen (p=0.051). In contrast, no differences between vaccine regimens were observed among HD patients. All participants maintained IgG titers well above the positivity threshold (7.1 BAU/mL); no individual had antibody levels below this cutoff.

Further analysis revealed no significant effect of demographic or clinical factors (sex, age ≥50 vs. <50 years, presence of diabetes or hypertension, dialysis vintage <1 vs. ≥1 year) on IgG levels in either HD patients or HCWs (p>0.05). However, there was a weak but statistically significant negative correlation between dialysis vintage and SARS-CoV-2 IgG levels in HD patients (Spearman’s ρ = -0.25; p=0.013). Linear regression showed that each additional year on dialysis reduced antibody levels by approximately 85 BAU/mL (β = -85.0; 95% CI: -151.5 to -18.5; p=0.013), although no specific dialysis-duration threshold could be identified. Thus, participant characteristics aside from vaccine type were not significantly associated with antibody responses.

When specifically examining vaccine regimens, HD patients showed no significant differences across CoronaVac-only, BNT162b2-only, or heterologous groups (p>0.05, **Figure 2B**). Conversely, vaccine type significantly influenced antibody levels among HCWs (p=0.007), with significantly lower IgG titers after CoronaVac-only vaccination compared to BNT162b2-only (p=0.008) and marginally compared to the heterologous regimen (p=0.051). No significant difference was observed between HCWs who received BNT162b2-only versus the heterologous regimen (p=0.395).

The total number of COVID-19 vaccine doses (which ranged from 2 to 4 in our cohort, with most having 3) was not significantly associated with IgG levels in either HD patients or HCWs (p>0.05), in other words, having an extra booster beyond the first booster did not show a measurable increase in antibody titer, although the numbers of participants with >3 doses were small. Additionally, in the HCWs group, we compared IgG levels among different job roles (physicians, nurses, and auxiliary health staff) to see if occupational exposure or other factors might play a role; no significant differences were observed (p>0.05).

Prior COVID-19 infection (before vaccination) had no significant effect on post-vaccination IgG levels in either group (HD or HCW) (p>0.05). In the HD group, only five patients had a history of COVID-19, and their antibody levels were comparable to other HD patients (p=0.121). In the HCW group, 22 individuals had a prior infection; their median IgG titer was slightly higher than those without infection, but the difference was not statistically significant after accounting for time since vaccination. This suggests that by the time of measurement (which was at least one month post-booster for all, and many months after natural infection for those individuals), the effect of natural infection (which can boost antibody levels) was no longer pronounced or was overshadowed by vaccine-induced immunity. **Figure 2A** shows that the distributions of IgG for previously infected vs not infected were largely overlapping in both HD patients and HCWs.

**Figure 3:**
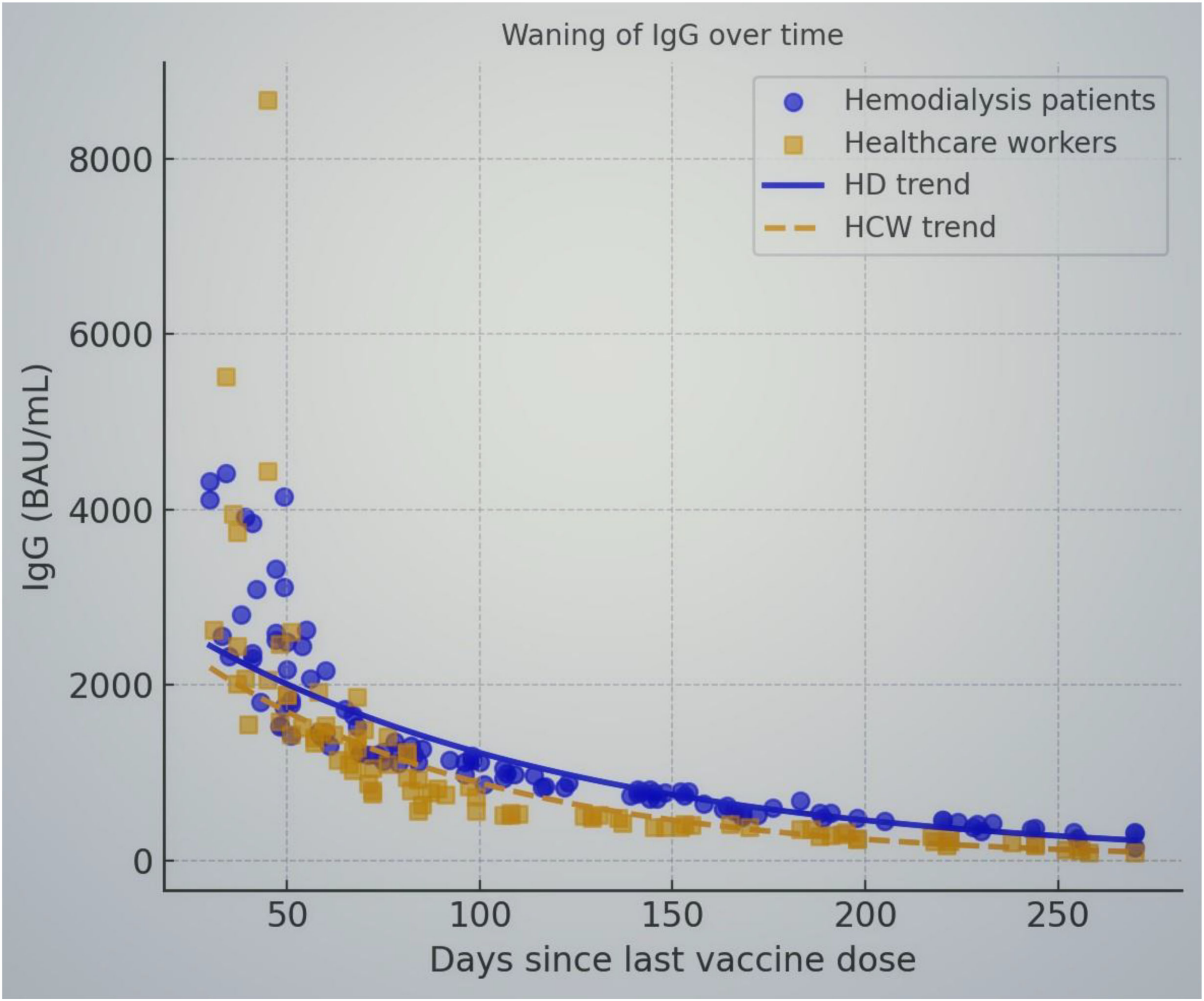
Waning of SARS-CoV-2 anti-spike IgG antibody levels over time since the last vaccine dose in HD patients (blue circles) and HCWs (orange squares). Each point represents an individual’s IgG level plotted against the number of days after their most recent vaccine dose. Solid blue and dashed orange lines indicate the fitted exponential decay trends for HD patients and HCWs, respectively. IgG levels decline over time in both groups, with higher titers observed soon after vaccination and a gradual decrease in subsequent months. The slopes of the decay curves are similar for HD patients and HCWs, suggesting that the rate of waning immunity is comparable. Even at ~9 months after vaccination, all participants maintained IgG levels above the positivity threshold (7.1 BAU/mL), though titers were substantially lower than early post-vaccination levels. This highlights the importance of booster doses to sustain immunity in both healthy and immunocompromised individuals.

Finally, we examined the relationship between time since last vaccine dose and IgG levels to assess waning immunity. Despite the overall robust responses, IgG levels declined significantly with increasing time post-vaccination in both groups. As illustrated in **Figure 3**, individuals sampled closer to their last vaccine dose tended to have higher antibody concentrations, whereas those sampled many months later had lower titers. In our cohort, the Spearman correlation between days since last dose and IgG titer was –0.77 (p<0.001) for HD patients and –0.61 (p<0.001) for HCWs, indicating a moderate to strong negative correlation. Both groups showed a similar pattern of waning: for example, at ~30–60 days after the booster, IgG levels were often very high (many in the several-thousand BAU/mL range), while by ~6–9 months after the booster, titers had declined, though still remained positive in all cases. **Figure 2** includes trend lines that demonstrate the exponential decay in antibody levels over time in each group. Importantly, even among those measured at the longest intervals (8–9 months post-vaccination), IgG levels in HD patients were comparable to those in HCWs at similar time points.

## Discussion

Our study demonstrates the immunogenicity and efficacy of COVID-19 vaccines in HD patients, who represent a population at substantially increased risk for severe COVID-19 outcomes. Initial concerns highlighted potential limitations in immune responsiveness due to uremia-associated immunosuppression. However, our results demonstrate robust antibody responses in dialysis-dependent individuals comparable to those observed in healthy HCWs, particularly following booster immunization. Unlike in HCWs, antibody responses among HD patients did not significantly differ based on the vaccine type, indicating potential flexibility in selecting vaccine strategies for this high-risk group. The observed decline in antibody titers over time emphasizes the necessity of timely booster vaccinations. Taken together, these findings reinforce current clinical guidelines advocating periodic booster administration to sustain adequate protective immunity and effectively mitigate COVID-19-related morbidity and mortality among patients undergoing HD.

In the present study, we found that HD patients, after completing a standard vaccination regimen (two doses plus a booster), achieved SARS-CoV-2 anti-spike IgG titers that were comparable to those of healthy HCWs. This is an encouraging finding, as it suggests that the humoral immune response in HD patients, at least in terms of antibody production, can be improved when vaccination is optimized. Our data showed no significant difference in mean or median IgG levels between HD patients and controls, and importantly, none of the HD patients failed to seroconvert (all had IgG above the positivity cutoff). This contrasts somewhat with earlier reports that a subset of dialysis patients had low or undetectable antibody responses after vaccination (24, 25). The difference may be attributable to the booster dose and the inclusion of mRNA vaccines: nearly 72% of our HD cohort received at least one dose of BNT162b2, which is known to elicit higher antibody titers than CoronaVac. Incontrast, CoronaVac induces higher CD4^+^ and CD8^+^ T‐cell responses to the structural protein than BNT162b2 (26, 27). Our findings align with those of Taheri et al. who reported substantial improvements in seroconversion rates in dialysis patients after a booster dose (28). It is also possible that dialysis patients have been exposed to subclinical infections or repeated antigenic stimulation during routine dialysis sessions, although we did not find prior PCR-confirmed infection to elevate antibody levels. The study was adequately powered to detect moderate differences in antibody responses between HD patients and HCWs. However, the observed antibody levels were highly comparable between the two groups, with no statistically significant difference. The confidence interval analysis further supported the absence of any clinically meaningful disparity. These findings suggest that dialysis status did not negatively impact the humoral response following COVID-19 vaccination.

We carefully considered potential confounding factors in interpreting the immune responses. The HD group was significantly older and had more comorbidities than the control group, which in general would predict a weaker vaccine response due to immunosenescence and chronic illness (29, 30). Indeed, older age and comorbidity (especially diabetes and cardiovascular disease) have been associated with reduced immunogenicity of COVID-19 vaccines in some studies (5–11, 31, 32). However, within our HD cohort, we did not observe an age-related drop in antibody levels, patients ≥50 years old had responses similar to those <50 years. Likewise, the presence of diabetes or hypertension did not show a measurable effect on titers. This could be because the booster dose helped overcome modest differences, or because our sample size was not large enough to detect small effects. It is noteworthy that despite being an older, comorbidity-burdened population, the HD patients responded well, demonstrating the vaccine’s efficacy in this high-risk group. Multivariate regression analyses further confirmed that HD status itself did not independently predict antibody responses after adjustment for potential confounders including age, sex, diabetes mellitus, hypertension, and dialysis vintage, supporting the robustness of our findings.

One key variable highlighted by our study is the type of vaccine regimen. In the HCWs, we observed clearly that two doses of CoronaVac yielded significantly lower antibody levels than two doses of BNT162b2, consistent with the known differences in immunogenicity between these platforms. Moreover, an mRNA booster after CoronaVac brought antibody levels up to nearly the same range as a full mRNA regimen, reflecting the benefit of heterologous boosting, as demonstrated in other studies (33, 34). Barin et al. similarly reported that anti-spike IgG concentrations one and three months after vaccination were highest in BNT162b2 recipients, intermediate with adenoviral vector vaccine (ChAdOx1), and lowest with CoronaVac; importantly, heterologous booster strategies significantly improved responses in those initially given CoronaVac (35). Our findings in HCWs mirror these patterns. However, among HD patients, vaccine-type differences were not statistically significant. Multiple comparisons were carefully controlled using Bonferroni adjustments, ensuring statistical rigor and minimizing the risk of Type I errors. This could suggest that the immune suppression in HD might blunt the advantages of the more potent vaccine, or alternatively, that even those HD patients who received the “weaker” vaccine (CoronaVac) eventually got an mRNA booster which compensated for it. In our HD group, many CoronaVac-only recipients actually had a third CoronaVac dose (since a subset received three inactivated doses by the time of the study). While their antibody levels were somewhat lower in absolute terms than the BNT162b2 recipients, the variation within groups was large and the sample size was limited, resulting in no detectable difference. It’s possible that with a larger sample, a trend favoring the mRNA vaccine in HD patients might emerge, but within our data, we can conclude that HD patients are capable of mounting strong IgG responses even to an inactivated vaccine, especially if boosted appropriately. Two-way ANOVA analysis revealed no significant interactions between participant group (HD vs. HCW) and vaccine regimen or prior infection status, highlighting the consistency of our findings across different patient subgroups.

Our study provides insight into waning immunity in both HD patients and healthy individuals. We conducted detailed exponential decay modeling to quantify antibody waning and statistically confirmed that there was no significant difference in antibody decay rates between HD patients and HCWs. We observed a clear decline in antibody levels over time since the last dose, with a moderate negative correlation between IgG titer and days post-vaccination. This is in line with the general understanding that antibody levels peak around 3–5 weeks after vaccination and then gradually decrease over the ensuing months (36, 37). The decline we noted (~4- to 5-fold decrease in median titer from 1–2 months to ~6 months) is comparable to what has been reported in non-immunocompromised cohorts. Notably, the rate of decline appeared similar in HD patients and controls. This suggests that while HD patients do not necessarily lose antibodies faster than others (once they’ve responded), they still face the issue of waning immunity and thus benefit from timely booster doses just as the general population does. In fact, given their higher risk if infected, maintaining adequate antibody levels through boosters is critical for HD patients. Our findings reinforce the recommendations for booster vaccinations in dialysis patients at intervals of about 6 months or as needed to counteract waning immunity.

It is important to highlight that prior SARS-CoV-2 infection (hybrid immunity) did not significantly elevate antibody levels in our analysis, which contrasts with some published studies. Glowińska et al. conducted a longitudinal study in dialysis patients and found that previous COVID-19 infection was associated with higher post-vaccination antibody concentrations and a longer duration of humoral immunity (38). They reported that patients with “hybrid immunity” (infection + vaccination) maintained detectable antibodies for longer periods than those who were never infected. In our study, the lack of a detectable infection effect could be due to several factors: the number of previously infected HD patients was very small (only five), limiting statistical power; and among the HCWs, many infections occurred early in the pandemic (before or between vaccine doses), so by the time of antibody measurement (which was post-booster) their infection-augmented titers may have normalized. Additionally, all previously infected participants still received full vaccination, which might have leveled the playing field, once vaccinated and boosted, the incremental difference provided by prior infection may diminish over time. It’s also possible that some “infection” cases in the control group were mild and did not dramatically boost antibody levels compared to the potent booster effect. Regardless, our data suggest that vaccination was the dominant contributor to antibody levels, and a prior infection was not necessary for achieving a strong response in HD patients or controls. This may indicate that a proper vaccine regimen can elicit high titers even in those without natural infection, which is reassuring for individuals who have avoided infection.

We found a positive correlation between dialysis duration and IgG levels in HD patients. One might expect longer dialysis vintage (which often correlates with older age and more comorbidities) to impair the immune response; however, in our cohort, those on dialysis longer had slightly higher titers. We speculate this could be related to those patients having had more cumulative antigen exposure (e.g., some may have received an additional vaccine dose or been exposed to COVID-19 in low levels during dialysis sessions). It might also be a survivor effect; patients who remain on dialysis longer could be those inherently more robust or responsive to vaccines. This correlation was weak and should be interpreted with caution, but it merits further exploration in larger studies. Despite this modest correlation (Spearman ρ= -0.25, p= 0.013), the clinical implications of dialysis vintage on vaccine responses remain uncertain and warrant further investigation.

Recent evidence supports that simultaneous administration of influenza and COVID-19 vaccines does not negatively impact safety profiles or immunogenic responses. In a study involving HCWs, simultaneous administration of the quadrivalent influenza vaccine (Flucelvax Tetra^®^) and the BNT162b2 mRNA COVID-19 vaccine (Comirnaty^®^) neither affected the safety nor was associated with a higher risk of SARS-CoV-2 breakthrough infection. The incidence of adverse events and breakthrough infections remained comparable between groups (39). Similarly, a prospective cohort study, evaluating co-administration of the Omicron BA.4/BA.5-adapted bivalent COVID-19 vaccine (Pfizer/BioNTech) with influenza vaccination (Influvac Tetra^®^, Abbott), found no significant increase in reactogenicity or decrease in immunogenicity (40). Geometric mean anti-spike IgG titers in the coadministration group were nearly equivalent to those receiving COVID-19 vaccination alone, further reinforcing the safety and effectiveness of coadministered vaccines. Moreover, the immunogenicity of COVID-19 vaccines in populations with potential immune dysfunction has been extensively studied, demonstrating robust antibody responses even in patients under immunosuppressive therapies. For instance, patients with psoriatic arthritis receiving tumor necrosis factor inhibitors (TNFi) achieved anti-SARS-CoV-2 IgG levels comparable to healthy controls following the BNT162b2 vaccine. Neither ongoing TNFi treatment nor short-term discontinuation of methotrexate substantially diminished the antibody response (15). Given all, these studies emphasize that the immunogenicity and effectiveness of COVID-19 vaccines are well-maintained even with simultaneous administration of other routine vaccines and among immunocompromised or frail patient groups.

The present study has some limitations. First, it is a cross-sectional assessment of antibody levels at a single time point for each participant. While we incorporated the time-since-vaccination in our analysis, longitudinal data following the same individuals would provide a more definitive picture of antibody kinetics (rise and fall) in HD patients versus controls. Second, our sample size, especially for subgroup analyses (e.g., HD patients with prior infection, or those in each vaccine category), was relatively small. This may have limited our ability to detect subtle differences. Third, we focused only on humoral immunity (circulating IgG antibodies). Neutralizing antibody activity and T-cell mediated immunity were not directly assessed. It is possible that HD patients could have impairments in T-cell responses despite having good antibody levels. Indeed, previous work indicates that cellular immunity may be more affected by uremia than humoral immunity (15, 16, 25, 26). Thus, comparable antibody levels do not necessarily guarantee equal protection, though higher antibody titers generally correlate with better neutralization of the virus. Lastly, the HCW control group, while convenient and relatively well-matched in basic demographics, is not a perfect representation of the general healthy population (they tended to be younger and predominantly female). However, this likely made our comparison more stringent, since HD patients were older and still fared well in the comparison. Notably, occupational roles (physicians, nurses, and other healthcare staff) did not significantly affect antibody responses, aligning with previous studies indicating no substantial differences based on occupational exposure.

In conclusion, our study demonstrates that HD patients, when appropriately vaccinated and boosted, can develop SARS-CoV-2 antibody responses that are as robust as those of healthy individuals. Despite older age and comorbidities, HD patients had no significant impairment in humoral immunity after COVID-19 vaccination. The use of an mRNA vaccine (or heterologous booster) appears particularly beneficial in maximizing antibody levels, and this strategy should be continued for dialysis patients to ensure optimal protection. We also confirm that antibody levels wane over time in HD patients, reinforcing the importance of booster doses at regular intervals to maintain immunity in this vulnerable group. While prior infection did not significantly augment vaccine responses in our cohort, other studies suggest hybrid immunity can be advantageou, and this area warrants further investigation with larger samples of previously infected dialysis patients.

Overall, these findings support current recommendations to prioritize COVID-19 vaccination (including booster shots) in HD patients. Continued surveillance of immune responses in HD patients is needed, including assessment of T-cell immunity and clinical protection, to fully understand vaccine efficacy in this population. Nonetheless, our data provide reassurance that *with the standard three-dose vaccination regimen, HD patients achieve a level of humoral immunity comparable to that of healthy people*. This robust antibody response likely contributes to the observed reduction in COVID-19 morbidity and mortality in vaccinated dialysis patients. Going forward, ensuring that HD patients receive timely booster vaccinations (preferably with mRNA vaccines) will be crucial to sustain their immunity, especially in the face of emerging variants and the natural waning of antibody levels. By maintaining high antibody titers and broad immunity, we can better protect dialysis patients from COVID-19 and its complications, thereby improving outcomes in this high-risk group.

## Data Availability

The raw data supporting the conclusions of this article will be made available by the authors, without undue reservation.
